# Cross-species discrimination of vocal expression of emotional valence by Equidae and Suidae

**DOI:** 10.1186/s12915-022-01311-5

**Published:** 2022-05-24

**Authors:** Anne-Laure Maigrot, Edna Hillmann, Elodie F. Briefer

**Affiliations:** 1 Institute of Agricultural Sciences, ETH Zürich, Universitätsstrasse 2, 8092 Zurich, Switzerland; 2 Division of Animal Welfare, Veterinary Public Health Institute, Vetsuisse Faculty, University of Bern, Länggassstrasse 120, 3012 Bern, Switzerland; 3 Swiss National Stud Farm, Agroscope, Les Longs-Prés, 1580 Avenches, Switzerland; 4 Animal Husbandry and Ethology, Albrecht Daniel Thaer-Institut, Faculty of Life Sciences, Humboldt-Universität zu Berlin, Philippstrasse 13, 10115 Berlin, Germany; 5 Centre for Proper Housing of Ruminants and Pigs, Federal Food Safety and Veterinary Office, Agroscope, Tänikon, 8356 Ettenhausen, Switzerland; 6 Department of Biology, Behavioral Ecology Group, Section for Ecology & Evolution, University of Copenhagen, 2100 Copenhagen Ø, Denmark

**Keywords:** Emotional contagion, Horses, Pigs, Przewalski’s horse, Vocal communication, Wild boars

## Abstract

**Background:**

Discrimination and perception of emotion expression regulate interactions between conspecifics and can lead to emotional contagion (state matching between producer and receiver) or to more complex forms of empathy (e.g., sympathetic concern). Empathy processes are enhanced by familiarity and physical similarity between partners. Since heterospecifics can also be familiar with each other to some extent, discrimination/perception of emotions and, as a result, emotional contagion could also occur between species.

**Results:**

Here, we investigated if four species belonging to two ungulate Families, Equidae (domestic and Przewalski’s horses) and Suidae (pigs and wild boars), can discriminate between vocalizations of opposite emotional valence (positive or negative), produced not only by conspecifics, but also closely related heterospecifics and humans. To this aim, we played back to individuals of these four species, which were all habituated to humans, vocalizations from a unique set of recordings for which the valence associated with vocal production was known. We found that domestic and Przewalski’s horses, as well as pigs, but not wild boars, reacted more strongly when the first vocalization played was negative compared to positive, regardless of the species broadcasted.

**Conclusions:**

Domestic horses, Przewalski’s horses and pigs thus seem to discriminate between positive and negative vocalizations produced not only by conspecifics, but also by heterospecifics, including humans. In addition, we found an absence of difference between the strength of reaction of the four species to the calls of conspecifics and closely related heterospecifics, which could be related to similarities in the general structure of their vocalization. Overall, our results suggest that phylogeny and domestication have played a role in cross-species discrimination/perception of emotions.

**Supplementary Information:**

The online version contains supplementary material available at 10.1186/s12915-022-01311-5.

## Background

Emotions are commonly defined as valenced (negative or positive) and intense but short reactions to an event of significance for the organism [1]. These reactions and their expressions are important in social species since they facilitate the regulation of social interactions [2]. They can be described using two main dimensions: their valence, which goes from negative (displeasure) to positive (pleasure), and their arousal (body activation or excitation), which goes from low (calm) to high (excited) [3]. In non-human animals, emotions can be assessed based on related physiological, behavioral, and cognitive changes [4]. When an emotion is expressed by an individual (e.g., through olfactory, visual or vocal signal), this information can be perceived by others (i.e., recognized and appraised), and sometimes trigger a similar emotion in the receiver of the signal; this automatic (i.e., without requiring effortful processing) transmission of emotional states is termed “emotional contagion” and has been defined as the first level of empathy (“the capacity to […] be affected by and share the emotional state of another”) [5]. Emotional contagion serves important functions not only in humans but also in other gregarious species. Indeed, this phenomenon can lead to the social spread and amplification of emotions (positive and negative) within a group of animals, which can then enhance group coordination and the strength of social bonds [2]. Emotional contagion has been suggested to be widespread in the animal kingdom and has been empirically shown to occur in some species, such as dogs (*Canis familiars* [6]), bonobos (*Pan paniscus* [7]), mice (*Mus musculus* [8]), and pigs (*Sus scrofa domestica* [9, 10]).

Individuals that meet regularly (i.e., that are familiar) or that are more phenotypically similar should discriminate or perceive each other’s emotions more easily [5, 11]. Since heterospecifics can also be familiar with each other (e.g., domestic species and humans, several species kept together, e.g., in zoos), discrimination, and possibly perception and contagion of emotions, could occur across species (“familiarity hypothesis”). Furthermore, since emotion expression has been suggested to be conserved throughout evolution [12], similarity in how closely related species express emotions could enhance emotion discrimination/perception (“phylogeny hypothesis” [13]). A third factor that could influence cross-species discrimination/perception of emotions is the process of domestication (“domestication hypothesis”); domestic animals may have been selected according to their similarity of emotion expression with humans, since such similarity should facilitate human-animal communication and the process of taming, or according to their ability to discriminate/perceive human’s emotion expression. Alternatively, species that were better in discriminating/perceiving human emotions could have tended to search for proximity to humans and be later domesticated [14].

Discrimination of human’s expression of emotions by animals has been shown to occur through facial cues in several domestic and captive species, such as dogs [15], horses (*Equus caballus* [16]), sheep (*Ovis aries* [17]), goats (*Capra hircus* [18]), giant pandas (*Ailuropoda melanoleuca* [19]), and chimpanzees (*Pan troglodytes* [20]). In contrast, evidence for discrimination/perception of human vocal expression of emotions is limited to dogs, horses, and cats [21]. These three domesticated species display cross-modal recognition (visual, i.e., facial expression, and vocal, i.e., emotional non-verbal vocalizations or speech) of human emotions [15, 22–24]. In addition, dog fMRI studies [25, 26] and horse behavioral experiments [27] suggest that dogs and horses can discriminate between positive and negative non-speech human vocalizations (e.g., growling and laughter). However, to our knowledge, no study has yet investigated the ability of non-human animals to discriminate emotions encoded in the vocalizations of other heterospecifics than humans. Knowledge from studies investigating, for example, discrimination/perception of emotions expressed in the vocalizations of closely related heterospecifics, is required to fully understand and decipher the mechanisms behind cross-species perception of emotions and therefore, the evolution of vocal expression of emotions.

**Fig. 1:**
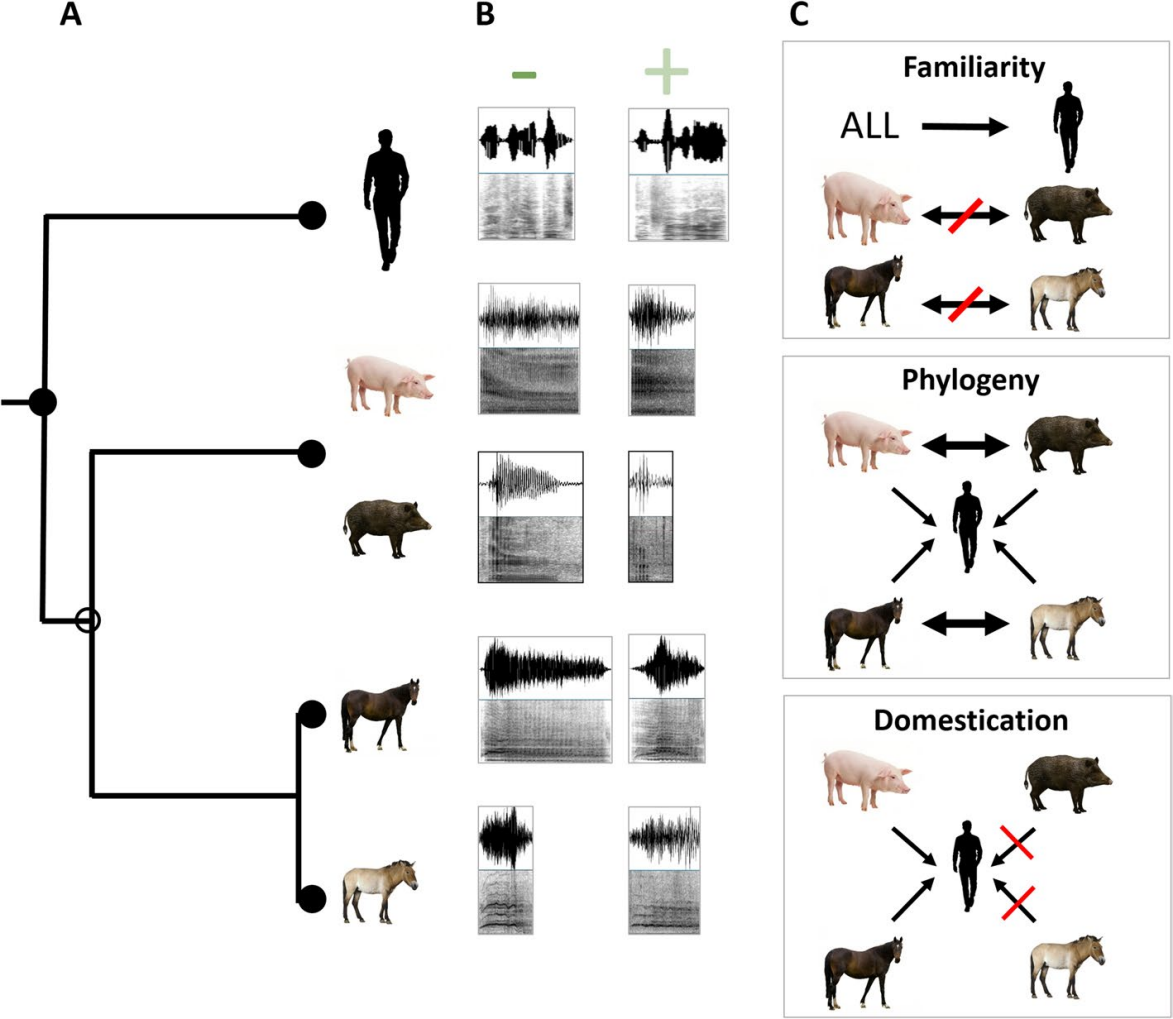
Hypotheses tested. **A** Phylogeny of the species played back [28]. **B** Examples of sounds produced in emotionally loaded negative and positive situations for each species, which were used in the playbacks (above, oscillogram; below, spectrogram). **C** Hypotheses tested in this study (non-exclusive): familiarity (all species recognize human emotions, as they are all exposed to human caretakers on a daily basis, while they should not recognize the emotions of the closely related species that they have never heard); phylogeny (closely related species recognize each other’s emotions better, or at least as well as human emotions); domestication (domestic species recognize human emotions, but wild ones do not)

In this study, we investigated the potential of the familiarity, phylogeny, and domestication hypotheses, to explain the occurence of the ability to discriminate vocal expression of emotions across species (Fig. 1). To this aim, we tested if domestic horses (*Equus callabus*), Przewalski’s horses (*Equus przewalskii*), domestic pigs (*Sus scrofa domestica*), and wild boars (*Sus scrofa*) discriminate between vocal expression of positive and negative valence in (a) unfamiliar conspecific calls, (b) closely related heterospecific calls (domestic horses to Przewalski’s horses and vice versa; pigs to wild boars and vice versa) and (c) human emotional speech (meaningless actor’s voices). We used, for all species, recordings for which the emotional valence associated with sound production was known and had been validated (using behavioral and/or physiological indicators for non-human species [29–32]; actors’ voices from the Geneva Multimodal Emotion Portrayal (GEMEP) Corpus for humans [33]).

Przewalski’s horses and wild boars are the closest relative of the domestic horses [34] and pigs [35], respectively, which currently live in the wild. Przewalski’s horses were, until recently, thought to be the only remaining “true” wild horse species. However, recent findings suggest that some ancestors of Przewalski’s horses could have been briefly domesticated in the Botai 5500 years ago before becoming feral [34]. Regarding Suidae species, wild boars were thought to be the principal genetic source of domestic pigs in Europe [35], but more recent findings suggest that the occurrence of an independent domestication of European wild boars did not occur [36]. Instead, European domestic pigs likely originate from cross breeding between European wild boars and near eastern domestic pigs, inducing a reciprocal gene flow that led to the disappearance of the near eastern domestic pigs’ genetic fraction [36].

**Fig. 2:**
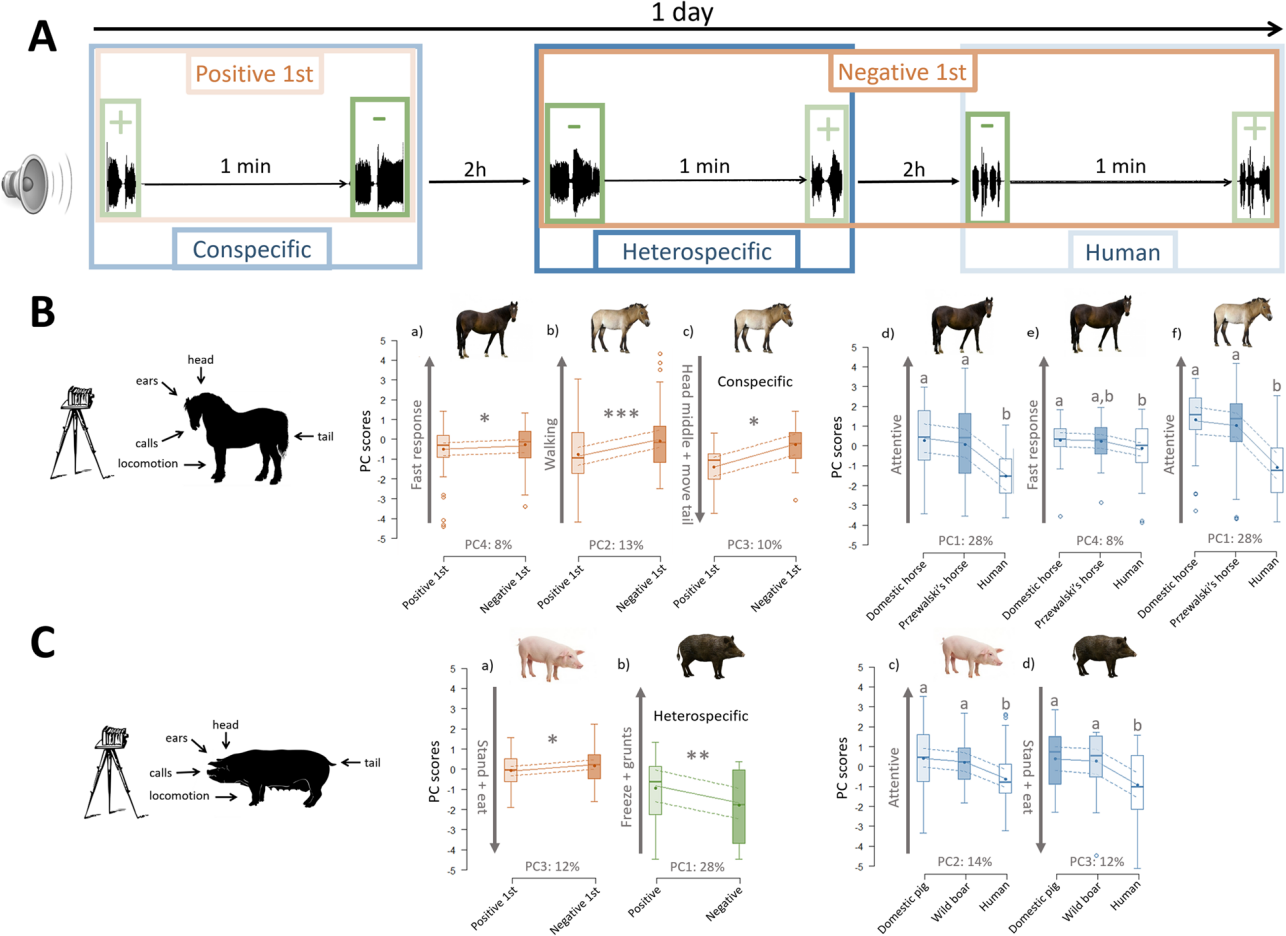
Experimental design and corresponding figures. **A** Experimental design showing three sessions of playbacks (one per species played) carried out over 1 day and the effects investigated (blue = species played; green = valence of the sound series; orange = valence of the first sound series of each session; the same color code is used for (**B**) and (**C**)). **B** Equidae’s responses to the playbacks: (a) domestic horse (PC4 scores) and (b) Przewalski’s horse (PC2 scores) responses as a function of the order in which positive and negative sounds (all species combined) were played; (c) Przewalski’s horse (PC3 scores) response as a function of the order in which positive and negative conspecific whinnies were played back; (d) domestic horse (PC1 scores), (e) domestic horse (PC4 scores) and (f) Przewalski’s horse (PC1 scores) responses as a function of the species played back (Tables 1 and 2). **C** Suidae’s behavioral responses to the playbacks: (a) domestic pig (PC3 scores) responses as a function of the order in which positive and negative sounds (all species combined) were played, (b) wild boar (PC1 scores) responses as a function of the valence of pig grunts played back, (c) domestic pig (PC2 scores) and (d) wild boar (PC3 scores) responses as a function of the species played back (Tables 3 and 4). Boxplots: the horizontal line shows the median, the box extends from the lower to the upper quartile and the whiskers to 1.5 times the interquartile range above the upper quartile or below the lower quartile, open circles indicate outliers and black circles the mean, the lines show the model estimates (continuous line) and 95% confidence intervals (dashed lines)

In order to differentiate between the familiarity and domestication hypothesis, the two wild species were studied in parks/zoos where the animals were familiar with humans, but not with the closely related domestic species. This set-up allowed us to establish clear predictions, according to our three non-exclusive hypotheses, about whether the animals should be able to discriminate or not between positive and negative vocalizations of conspecifics, closely related heterospecifics and humans (Fig. 1). Each sound treatment consisted of a short series of positive sounds (2–6 sounds depending on the species and hence the sound duration), followed after 1-min silence interval by a short series of negative sounds, or vice-versa, in a random order (Fig. 2A). Each sound consisted of one animal call, or 2 s of human voice. For both taxa (Equidae and Suidae), all sounds were played back at the same intensity and thus differed only in terms of species played (conspecifics, closely related heterospecifics or humans), valence (positive or negative), and valence order (positive or negative sounds played first).

## Results

### Do Equidae and Suidae discriminate between conspecific and heterospecific vocalizations of opposite valence?

Both species of Equidae reacted more strongly (domestic horses responded faster and Przewalski’s horses spent more time walking and less time standing) to the playbacks when the first vocalizations broadcasted were negative compared to positive, regardless of whether these vocalizations were produced by conspecifics, closely related heterospecifics or humans (effect of the valence of the first sound series on PC4 and PC2, respectively; Fig. 2B (a, b); Tables 1 and 2). In addition, Przewalski’s horses spent less time with the head in the middle and displayed less tail movements, suggesting more attentive behavior, when they first heard conspecific negative calls than conspecific positive calls (effect of the valence of the first sound series on PC3; Tukey’s honest significant difference (HSD) test: *z* = −2.96, *p* = 0.036; Fig. 2B (c)). This was not the case when they heard domestic horse calls or human voices (effect of the valence of the first sound series on PC3; Tukey’s HSD test: *p* ≥ 0.41 for both).

**Table 1:** Equidae loadings. Loadings of the behaviors on the principal components with eigenvalue > 1 (PC1–PC4) extracted from the principal component analysis conducted on the Equidae responses (domestic and Przewalski’s horses combined). Behaviors with a loading ≥ *r* = l0.45l appear in bold

PC1		PC2		PC3		PC4	
1st behavior	−0.27	1st behavior	0.12	1st behavior	0.31	1st behavior	−0.61
1st movement	0.11	1st movement	0.36	1st movement	−0.11	1st movement	−0.44
**Looking at the loudspeaker**	**0.75**	Looking at the loudspeaker	−0.32	Looking at the loudspeaker	0.06	Looking at the loudspeaker	−0.09
**Standing**	**−0.50**	**Standing**	**−0.73**	Standing	−0.23	Standing	−0.12
**Walking**	**0.53**	**Walking**	**0.74**	Walking	0.14	Walking	0.05
**Head movements**	**0.48**	Head movements	0.04	Head movements	−0.38	Head movements	−0.09
**Head high**	**0.76**	Head high	−0.25	Head high	0.19	Head high	0.21
Head in the middle	0.08	Head in the middle	0.28	**Head in the middle**	**−0.75**	Head in the middle	−0.09
Ear movements	0.35	Ear movements	−0.30	Ear movements	−0.25	Ear movements	0.27
**Ear on the sides**	**−0.66**	Ear on the sides	0.19	Ear on the sides	−0.17	Ear on the sides	0.42
**Ear forwards**	**0.81**	Ear forwards	−0.24	Ear forwards	0.11	Ear forwards	−0.27
Tail movements	−0.12	Tail movements	0.03	**Tail movements**	**−0.57**	Tail movements	−0.28
Tail low	−0.01	Tail low	−0.37	Tail low	−0.08	Tail low	−0.25
**Eating**	**−0.80**	Eating	−0.07	Eating	0.28	Eating	−0.15
**Eigenvalue**	**1.97**	**Eigenvalue**	**1.34**	**Eigenvalue**	**1.20**	**Eigenvalue**	**1.07**
**Variance**	**28%**	**Variance**	**13%**	**Variance**	**10%**	**Variance**	**8%**

**Table 2:** Equidae statistics. Statistical results (*P-*values extracted from linear mixed-effects models by parametric bootstrap) for the Equidae (significant *p*-values appear in bold)

**Domestic horse**						
**PC**	Species played * Valence of the sound series	Valence of the sound series * Valence of the 1st sound series	Species played * Valence of the 1st sound series	**Valence of the sound series**	**Species played**	**Valence of the 1st sound series**
**PC 1**	0.35	0.24	0.50	0.39	**≤0.001**	0.93
**PC 2**	0.27	0.54	0.66	0.46	0.32	0.16
**PC 3**	0.45	0.68	0.28	0.19	0.41	0.25
**PC 4**	0.06	0.10	0.11	0.18	**0.01**	**0.038**
**PC 5**	0.63	0.74	0.76	0.09	0.34	0.98
**Przewalski’s horse**						
**PC**	Species played * Valence of the sound series	Valence of the sound series * Valence of the 1st sound series	Species played * Valence of the 1st sound series	**Valence of the sound series**	**Species played**	**Valence of the 1st sound series**
**PC 1**	0.26	0.10	0.19	0.30	**≤0.001**	0.81
**PC 2**	0.75	0.62	0.94	0.10	0.28	**≤0.001**
**PC 3**	0.55	0.57	**0.005**	0.27	0.06	0.90
**PC 4**	0.54	0.39	0.84	0.54	0.47	0.82
**PC 5**	0.44	0.38	0.96	0.67	0.38	0.18

Domestic pigs reacted more strongly (spent less time eating and standing, i.e., not walking or running, and reacted faster) to the playbacks when the first vocalizations played were negative compared to positive, irrespectively of the species producing the sounds (effect of the valence of the first sound series on PC3; Fig. 2C (a); Tables 3 and 4). This suggests that pigs, in the same way as domestic and Przewalski’s horses, can discriminate between positive and negative sounds of conspecifics, closely related heterospecifics and humans. By contrast, wild boars did not react differently to positive and negative vocalizations of wild boars or human voice (effect of the valence of the sound series on PC1; Tukey’s HSD test: *p* ≥ 0.15 for both), but to positive and negative vocalizations of pig calls; they moved their head more often, spent more time with their ears on the sides, with the tail high and standing, and produced more grunts, when positive pig calls were played compared to negative ones (*z* = −3.27, *p* = 0.001, Fig. 2C (b)).

**Table 3:** Suidae loadings. Loadings of the behaviors on the principal components with eigenvalue > 1 (PC1–PC4) extracted from the principal component analysis conducted on Suidae responses. Behaviors with a loading ≥ *r* = l0.45l appear in bold

PC1		PC2		PC3		PC4	
1st behavior	−0.15	1st behavior	−0.20	1st behavior	−0.49	1st behavior	0.59
1st vocalization	0.44	1st vocalization	0.01	1st vocalization	−0.07	1st vocalization	0.04
1st movement	0.29	1st movement	0.36	1st movement	−0.41	1st movement	0.41
**Standing**	**0.56**	**Standing**	**0.47**	**Standing**	**−0.53**	Standing	−0.26
Walking	0.44	**Walking**	**−0.69**	Walking	0.29	Walking	0.06
**Head movements**	**0.83**	Head movements	−0.04	Head movements	0.12	Head movements	0.08
**Head on the middle**	**0.51**	**Head on the middle**	**0.54**	Head on the middle	0.34	Head on the middle	0.27
Head low	0.18	Head low	0.00	Head low	−0.07	**Head low**	**−0.66**
**Ears on the sides**	**0.83**	Ears on the sides	−0.04	Ears on the sides	−0.29	Ears on the sides	−0.23
**Tail high**	**0.69**	Tail high	−0.12	Tail high	0.33	Tail high	0.11
**Grunt**	**0.59**	Grunt	−0.16	Grunt	0.18	Grunt	0.16
Eating	0.19	**Eating**	**−0.64**	**Eating**	**−0.59**	Eating	−0.04
**Eigenvalue**	**1.83**	**Eigenvalue**	**1.27**	**Eigenvalue**	**1.22**	**Eigenvalue**	**1.09**
**Variance**	**28%**	**Variance**	**14%**	**Variance**	**12%**	**Variance**	**10%**

**Table 4:** Suidae statistics. Statistical results (*P*-values extracted from linear mixed-effects models by parametric bootstrap) for the Suidae (significant *p*-values appear in bold)

**Pigs**						
**PC**	Species played * Valence of the sound series	Valence of the sound series * Valence of the 1st sound series	Species played * Valence of the 1st sound series	**Valence of the sound series**	**Species played**	**Valence of the 1st sound series**
**PC 1**	0.46	0.45	0.13	0.36	0.49	0.07
**PC 2**	0.87	0.84	0.46	0.38	**0.005**	0.17
**PC 3**	0.23	0.94	0.55	0.37	0.49	**0.028**
**PC 4**	0.16	0.39	0.76	0.93	0.70	0.09
**PC 5**	0.78	0.94	0.87	0.46	0.59	0.79
**Wild boars**						
**PC**	Species played * Valence of the sound series	Valence of the sound series * Valence of the 1st sound series	Species played * Valence of the 1st sound series	**Valence of the sound series**	**Species played**	**Valence of the 1st sound series**
**PC 1**	**0.025**	0.13	0.74	0.26	0.49	0.84
**PC 2**	0.25	0.85	0.20	0.23	0.71	0.10
**PC 3**	0.85	0.79	0.17	0.83	**0.008**	0.88
**PC 4**	0.57	0.82	0.87	0.27	0.12	0.11
**PC 5**	0.59	0.38	0.95	0.22	0.26	0.34

### Do Equidae and Suidae respond differently to conspecific and closely-related heterospecific calls?

All four species tested reacted similarly to the session of calls of both conspecific and closely related heterospecifics, and less markedly to human voice. Indeed, both species of Equidae spent less time with the ears pointed forward, more time eating, less time with the head high and looking at the loudspeaker, more time with the ears on the side, less time walking, and more time standing and made less head movements when we played human voices than when we played calls of Equidae (effect of the species played on PC1; Tukey’s HSD test: *p* ≤ 0.001 for both; Fig. 2B (d, f)). By contrast, these behaviors did not differ between playbacks of domestic and Przewalski’s horses (effect of the species played on PC1; Tukey’s HSD test: *p* ≥ 0.75 for both; Fig. 2B (d, f)). Additionally, domestic horses were slower to respond when hearing human voices compared to conspecifics’ calls (effect of the species played on PC4; Tukey’s HSD test: *z* = 2.17, *p* = 0.047), while this behavior did not differ between playbacks of domestic and Przewalski’s horse, nor between playbacks of Przewalski’s horse and humans (*p* ≥ 0.16 for both; Fig. 2B (e)). Similarly, when listening to human voice compared to the calls of both Suidae species, pigs spent more time walking and eating, and less time with their head in the middle and standing (effect of the species played on PC2; Tukey’s HSD test: *p* ≤ 0.035 for both, Fig. 2C (c)), and wild boars spent more time eating and standing, and were slower to react (effect of the species played on PC3; *p* ≤ 0.010 for both), while these behaviors did not differ between playbacks of pigs and wild boars (*p* ≥ 0.80 for both; Fig. 2C (c, d)).

## Discussion

In order to decipher the factors influencing cross-species discrimination of vocal expression of emotions, we tested whether four species of domestic and wild ungulates (domestic horses, Przewalski’s horses, domestic pigs, and wild boars) were able to discriminate between vocalizations produced under emotional contexts of opposite valence in conspecific calls, closely related heterospecific calls, and human voices. The potential factors that we investigated were familiarity with the species, domestication, and phylogeny (Fig. 1). We found that, except for wild boars, all species tested reacted differently (e.g., shorter latency to react, less time spent eating) when the first calls of the series were negative compared to positive, independently of the species played back, suggesting some abilities to discriminate between sounds of opposite emotional valence across species, including in human voices. Wild boars on the other hand showed stronger reactions (e.g., more head movements and more calls) when positive pig calls were played compared to negative ones, while they did not respond differently to positive and negative calls from any other species, including their own. Finally, we observed that all four species reacted less and were less attentive when human voice was played, compared to conspecific and closely related heterospecific calls, independently of the valence. In the rest of the discussion, we will discuss which of our three hypotheses (familiarity, domestication or phylogeny) these results fit best.

The familiarity hypothesis predicts that animals should distinguish the emotional valence better in the vocalizations of familiar than unfamiliar species, following a process of learning with repeated exposure (Fig. 1). We indeed found that Przewalski’s horses reacted in a way that suggested more attentive behaviors, when the first calls of the series were conspecific negative calls compared to positive ones, while these behaviors did not vary with the valence order of other species played back. This suggests that Przewalski’s horses are able to discriminate between vocalizations of opposite valence produced by the species with which they are the most familiar (i.e., their own species). However, we also found that other behaviors in these horses (e.g., time spent walking), as well as the behavior of domestic horses and pigs, differed depending on the valence of the first calls of the playback series, regardless of which species was played. This implies that all three species are able to discriminate between positive and negative vocalizations of conspecifics and humans, with whom they are familiar, but also vocalizations of closely related heterospecifics, which they have never heard. Furthermore, wild boars responded differently only to positive versus negative calls of domestic pig, which is the species they were the least familiar with in our study. Therefore, the familiarity hypothesis does not seem to be supported by our results. In humans, familiarity with a species has been shown to improve recognition of its emotion or of the context of production associated with vocal production for cats [37], pigs [38], chimpanzees, and tree shrews [13], while mixed results have been found for dogs [39, 40].

The domestication hypothesis predicts that domestic animals should discriminate/perceive human emotional states better than wild species, following a selection (likely unconscious) during domestication, of individual animals with this ability (Fig. 1). Accordingly, our results suggest that both domestic species (horses and pigs) are able to discriminate between positive and negative human meaningless speech. Also in accordance with this hypothesis, wild boars do not seem able to do so. However, our results suggest that Przewalski’s horses, which have not been domesticated [41] or only briefly [34], are able to discriminate between human vocalizations of opposite valence as well. Therefore, the domestication hypothesis could explain the results we obtained in Suidae, but not in Equidae. To our knowledge, our study provides the first test for the effect of domestication on human-animal discrimination of vocal expression of emotion, in which both domestic and closely related wild species are included.

The phylogeny hypothesis predicts that, due to a conservation of indicators of emotions throughout evolution, animals should discriminate/perceive the valence encoded in the vocalizations of conspecifics better, or at least as well, as in those of closely related heterospecifics. In addition, they should discriminate/perceive emotions encoded in the vocalizations of closely related heterospecifics better, or at least as well, as in human voice (Fig. 1) [12]. Our results suggest that, in accordance with the phylogeny hypothesis, domestic horses, Przewalski’s horses, and pigs are able to discriminate between positive and negative vocalizations of conspecifics and heterospecifics, but also human meaningless speech. Moreover, the response of Przewalski’s horses was more pronounced when hearing conspecific negative calls first compared to positive calls, which was not the case when hearing other species, and is also in accordance with the phylogeny hypothesis. However, surpringly, wild boars reacted differently to positive and negative calls of pigs but not of their own species. Therefore, the phylogeny hypothesis might explain the results we obtained in Equidae, but not in Suidae. This is in accordance with recent studies showing that domestic horses display cross-modal recognition (visual and vocal) of human emotions [22, 23], and discriminate between negative and positive human nonverbal vocalizations [27]. In humans, this hypothesis has not been verified. Indeed, it has been shown that phylogeny is not the main factor influencing human perception of emotions in animal vocalizations [13] and that humans are able to perceive the level of emotional arousal (i.e., bodily excitation [3]) in the vocalizations of a wide range of taxa [42].

We found that wild boars showed a different reaction as a function of the valence only when pig sounds were broadcast. Interestingly, the behavior that these animals displayed when positive pig calls were played, such as tail high, standing (“freezing”), and grunts, have been shown to indicate negative emotions in domestic pigs (e.g., [9, 29, 43]). If these behaviors also indicate negative emotions in wild boars, this would suggest that wild boars experienced negative emotions when hearing positive pig calls. This could be due to the opposite way in which wild boars and domestic pigs express their emotions through vocalization. Indeed, salient vocal parameters, such as the frequency of formants (resonance frequencies), decrease from negative to positive valence in wild boars, while they increase in pigs [29, 32]. We hypothesize that this difference between domestic pig and wild boar vocal expression of emotional valence could be a by-product of the documented changes in behavioral response toward humans and their environment that have occurred during the domestication process (e.g., reduction in the size of structures within the limbic system, resulting in an overall reduction of emotional reactivity [44]). Alternatively, behavioral indicators of valence might differ between the two species, in the same way as their vocal indicators, and the reaction of wild boars to positive pig calls might in fact not indicate negative emotions.

Finally, all species reacted as strongly to the calls of the closely related heterospecifics as to the calls of their conspecifics, while they displayed weaker responses to humans. This suggests that the acoustic structure of domestic horse and Przewalski’s horse whinnies, as well as of domestic pig and wild boar grunts, is similar enough to trigger a species-specific response. Indeed, it has been shown that the structure of Przewalski’s horse whinnies resembles the structure of domestic horse whinnies, since both contain two fundamental frequencies, suggesting biphonation [30, 31]. Regarding Suidae, the two species also produce grunts that are similar in structure, with a low fundamental frequency and three main salient formants [29, 32].

It should be noted that, although we played the same set of sounds to related domestic and wild species and designed our experiments and planned our analyzes with the aim of maximizing similarity between how they were tested, these species did not only differ in the domestic process they had been through, but also in other aspects, such as how habituated to humans they were (e.g., domestic horses had much closer contact to humans than Przewalski’s horses), and the group sizes in which they were tested (for practical reasons, wild species had to be tested within a group (range = 2 to 12 individuals), while domestic species were always tested in pair). All these aspects could have had an influence on the reaction of the animals. For instance, responses may have been stronger in larger groups, since the size of the group might influence social transmission, despite our attempt to control for these differences between groups by using a repeated measure design and adding the identity of the group as a random effect in our models. On the other hand, our approach of combining wild and domestic species in the same principal component analysis might have dampened within-species differences in responses to the playbacks and is therefore relatively conservative. Overall, we believe that obtaining significant effects despite such variation between locations, animals, and settings, suggests that our results are robust and reproducible [45]. Yet, we also acknowledge that, since our experiment was aimed at testing the discrimination of emotional vocalizations within and across several species, our design simultaneously included many factors (species, valence, valence order), making it complex. Further research aimed at validating these findings with further domestic and wild species, more indicators of emotions (e.g., infra-red thermography) and in different environments, would be very valuable.

## Conclusions

To conclude, domestic horses, Przewalski’s horses, and pigs were found to distinguish vocal indicators of valence in all the species we played back, while wild boars only did so in pig calls. The results we obtained in Equidae could thus be explained by the phylogeny hypothesis, while the responses of Suidae are more in accordance with the domestication hypothesis. Whether cross-species discrimination/perception of vocal expressions of emotions is rendered possible by a conservation of vocal indicators of emotional valence throughout evolution (phylogeny hypothesis) or by a selection of individual animals that were able to discrimination/perceive human vocal expression of emotions better than others throughout the domestication process seems to depend on the Family (Equidae versus Suidae) or Order (Perissodactyla versus Artiodactyla). Further studies could investigate if these results hold when considering more species and Families, and also other channels (e.g., visual, olfactory), as well as how integrating different modalities affects cross-species perception of emotions. Our results also suggest that the valence of human voice can have an impact on the emotional states of domestic and captive animals, and stress the need to further assess if, how, and when does human-animal vocal emotional contagion occur, using additional indicators of emotions not used in this study (e.g., physiological or cognitive indicators).

## Methods

### Subjects and management conditions

The study was conducted between April and August 2015 on four different species: domestic horses, Przewalski’s horses, domestic pigs, and wild boars. Regarding the domestic species, we tested twelve pairs of domestic horses from 14 different breeds housed in four private riding farms in Switzerland, and twelve pairs of domestic pigs (Swiss Large White breed) from two different batches kept at the Agroscope Research Station (Taenikon, Switzerland). The horses were all born from different parents and did not, to our knowledge, share any immediate parentage. The domestic pigs were born from 11 different mothers who were artificially inseminated with semen from a small number of breeding boars. The domestic horses were all kept in single boxes or boxes with paddocks and had regular access to a field (10-12 h per day), and the pigs were kept together with other pigs of the same batch and age in one pen with deep straw. Regarding the wild species, we tested twelve groups of Przewalski’s horses (2–24 individuals per group) in six wildlife parks in France and Switzerland, as well as ten groups of wild boars in ten wildlife parks in France and Switzerland. Today’s Przewalski’s horses all descend from 9 to 12 breeding males among the 31 individuals that were held captive at the time the species went extinct from the wild around 1969. However, the groups tested in different parks did not, to our knowledge, share any immediate parentage. The groups of boars were all of various origins and, to our knowledge, did not share any immediate parentage either. The Przewalski’s horses were housed either in paddocks (for six groups; 70–150 m^2^) with an access to an adjacent field or in a large enclosure (400–700 hectares), and the wild boars were housed in adapted enclosures with an access to a shelter (about 20 m^2^).

### Playback treatments

We used a unique set of recordings obtained during our previous studies, for which the emotional states of the producers were known and had been validated using behavioral (domestic and wild species, e.g., head, ears, tail and body position and movements, call rate) and/or physiological indicators (domestic species; heart rate and heart-rate variability, respiration rate, and skin temperature) [29–32]. To obtain these recordings, the domestic species were placed in contexts assumed to induce positive and negative emotions, while the wild species were recorded opportunistically during naturally occurring emotional contexts. The domestic horses were recorded in four contexts: reunion (positive) and separation (negative) with either all or only one group member [30]. For the domestic pigs, two contexts were used: in pair with food, water, and toys (positive), and during isolation (negative) [29]. The Przewalski’s horses and wild boars were recorded during anticipation of a food reward and affiliative interactions (positive), as well as agonistic interactions and social separation (only Przewalski’s horses) (negative) [31, 32]. Analyses of these recordings revealed that the acoustic structure of vocalizations (whinnies for Equidae and grunts for the Suidae) differed according to the emotional valence of the contexts [29–32]. For the human recordings, we used voices of actors from a validated database (Geneva multimodal emotion portrayal) playing joy and amusement (positive), as well as anger and fear (negative) [33].

For both Equidae and Suidae, the domestic and wild species were tested with the same set of sounds. Each pair or group of Equidae (domestic and Przewalski’s horses) was tested (i.e., repeated-measure design) once with the following six treatments grouped in three sessions; positive and negative whinnies from domestic horses (session 1), positive and negative whinnies from Przewalski’s horses (session 2), and human voice representing a positive emotion (joy and amusement) and a negative emotion (fear and anger) (session 3; Fig. 2A). Similarly, each pair or group of Suidae (pigs and wild boars) was tested with the following 6 treatments grouped in 3 sessions: positive and negative grunts from domestic pigs (session 1), positive and negative grunts from wild boars (session 2), and human voice representing a positive emotion (joy and amusement) and a negative emotion (fear and anger) (session 3; Fig. 2A). Such repeated-measure design allowed us to consider each pair or group as their own control and avoid the use of an extra control treatment, hence minimizing risks to trigger a fading of response strength over repeated exposition, which is a common issue with playback experiments [46].

Domestic and Przewalski’s horse treatments were prepared as follows; each playback session consisted of a series of two positive whinnies (with 2 s of silence interval) followed by a series of two negative whinnies of the same individual (with 2 s of silence interval) after 1 min of silence, or vice versa (i.e., two negative whinnies and two positive ones). Preparation of sessions involved selecting the two best quality whinnies (low level of background noise) from 12 domestic horses (6 males and 6 females) and 6 Przewalski’s horses (3 males and 3 females) that had vocalized the most in our previous studies [30, 31]. The number of horses used to prepare the playbacks was maximized so that each domestic horse was used for no more than two groups of Przewalski’s horses or pair of domestic horses and each Przewalski’s horse was used for no more than three groups of Przewalski’s horses or pair of domestic horses (each domestic horse was played to 1.09 ± 0.3 groups of Przewalski’s horses or pair of domestic horses; range = 1–2; each Przewalski’s horse was played to 2 ± 0.89 groups of Przewalski’s horses or pair of domestic horses; range = 1–3). In the cases (*n* = 12/24 sequences) where it was not possible to obtain two different good quality whinnies from the same horse to prepare a sequence, the same whinny was repeated twice.

Pig and wild boar treatments were prepared as follows; each playback session consisted of a series of four to six (depending on the duration of the calls and to reach 5 s per sequence) positive grunts (with 0.5 to 2 s of silence interval between each grunt) followed by a series of four to six negative grunts of the same individual (with 0.5 to 2 s of silence interval between each grunt) after 1 min of silence, or vice versa (i.e., four to six negative grunts and four to six positive ones). Preparation of sessions involved selecting the four to six best quality grunts (low level of background noise) from 12 domestic pigs (6 males and 6 females) and 12 wild boars (6 males and 6 females) that had vocalized the most in our previous studies [29, 32]. The number of animals used to prepare the playbacks was maximized so that each domestic pig and each wild boar was used for no more than one group of wild boars or pair of pigs. In the only case (*n* = 1/24 sequences) where it was not possible to obtain enough different good quality grunts to prepare a sequence, the same grunt was repeated for a maximum of two times.

In order to match the duration and rate of animal sequences, the human treatments were prepared in the following way; each playback session consisted of a series of two times 2 s of positive meaningless speech (with 2 s of silence interval) followed by a series of two times 2 s of negative meaningless speech of the same actor (with 2 s of silence interval) after 1 min of silence, or vice versa (two times 2 s of negative voice and two times 2 s of positives ones). The number of human actors (*n* = 10 actors, 5 males and 5 females) used to prepare the playbacks was maximized so that each actor was used for no more than two pairs or groups of animals (each voice was played to 1.2 ± 0.4 pair of domestic horses or pigs, or groups of Przewalski’s horses or wild boars; range = 1–2).

For each treatment described above, the short silence interval between the sounds associated with positive and negative valence (1 min) was necessary for practical reasons when testing the wild species in large parks, in order to avoid losing sight of the animals and having to run these two treatments in conditions that differed too much. All individual vocalizations and sequences (domestic horses, Przewalski’s horses, pigs, wild boars, and humans) were scaled to the same relative absolute peak amplitude of 0.99. They were prepared using Praat v.5.3.41.

### Playback procedure

Sounds were broadcast with an AmpliVox SW800 Titan Wireless Portable PA System (frequency response: 40 Hz to 20 Khz), connected to a laptop where the sounds were stored in WAV format, at a sampling rate of 44.1 kHz and a bit rate of 705 kbps. Sounds were played at an intensity estimated to be normal for the animals, and for each Family, the intensity of the six treatments was homogenized (93.59 ± 0.71 dB for domestic horse calls; 93.16 ± 1.63 dB for Przewalski’s horse calls; 91.26 ± 3.65 dB for human voices to Equidae; 86.62 ± 6.92 dB for domestic pig calls; 87.19 ± 5.89 dB for wild boar calls; 89.08 ± 5.28 dB for human voices to Suidae; measured at 1 m using a sound level meter, C weighting, SoundTest-Master, Laserlinerer, UK). Since pairs of domestic animals were isolated from the rest of the group for the test, all the horses that were not usually housed by pair and hence could not be tested directly in their home pens (*n* = 20), and all the pigs, were habituated to the procedure beforehand. The habituation procedure consisted in being led to the test arena by pair for 10 min during three consecutive days. For the playbacks, the loudspeaker was placed out of view and between 3 and 25 m away from the animals. This distance was similar between sessions for each group of animals tested. After setting-up the recording material, we waited for all individuals to return to normal behaviors before broadcasting the first treatment of each playback session. For every pair or group, the three playback sessions were conducted on the same day (Fig. 2A). The next playback sessions started 2 h later, in order to prevent habituation. The order of the sessions (i.e., species played), the order of the valence of the two sound series within each session (i.e., if a series of negative of positive sound was played first) as well as the sex of the individual used to prepare the playback sequences was set randomly.

### Behavioral measures

All tests were filmed using a Sony Camcorder HDR-PJ240ES by an experimenter situated away from the loudspeaker. The behavioral parameters described in Table 5 were scored from the videos of the tests using The Observer XT v.11.5 (Noldus), for each treatment (positive and negative), continuously for 50 s following the beginning of the first vocalization of each series played back. We chose to score all the behaviors that were clearly visible on the video, that are commonly included in studies focusing on animal emotions [4], and which were displayed by both the domestic and the closely related wild species to allow us to compare the responses of the related species [29–32]. In order to obtain matched sample sizes for the four species, and since the domestic species were tested in pairs, while the wild ones were tested in groups (range = 2 to 24 individuals per group), we scored the behavior of the two domestic horses and pigs in each pair, and of two randomly selected focal Przewalski’s horses and wild boars within each group among those clearly visible on the video. Random selection was achieved by attributing a number to every visible animal and using a program in R software v.3.2.1 to select two numbers randomly. For each group of wild species, the same two randomly chosen individuals were scored throughout the all treatments.

**Table 5:** Ethogram. Description of the behavioral parameters that were scored. Bold parameters indicate those that were kept for the analyses (i.e., performed by > 50% of the animals of each species)

	Abbreviation	Family	Description
**Latency of reaction**	**1**^**st**^ **behavior**	Both	Latency between the beginning of the first sound broadcasted and the animal’s first behavioral reaction
	**1**^**st**^ **vocalization**	Both	Latency between the beginning of the first sound broadcasted and the animal’s first vocalization
	**1**^**st**^ **movement**	Both	Latency between the beginning of the first sound broadcasted and the animal’s first movement
**Reaction toward the loudspeaker**	Approaching the loudspeaker	Both	Proportion of time spent approaching the loudspeaker
	**Looking at the loudspeaker**	Both	Proportion of time spent looking at the loudspeaker
	Avoiding the loudspeaker	Both	Proportion of time spent walking away from the loudspeaker
**Movements**	**Standing**	Both	Proportion of time spent standing
	**Walking**	Both	Proportion of time spent walking
	Trotting	Equidae	Proportion of time spent trotting
	Cantering	Equidae	Proportion of time spent cantering
	Running	Suidae	Proportion of time spent running
**Head**	**Head movements**	Both	Number of head movement per minute
	**Head high**	Both	Proportion of time spent with the line of the eyes above the tip of the shoulder
	**Head on the middle**	Both	Proportion of time spent with the line of the eyes at the same level as the shoulder tip
	**Head low**	Both	Proportion of time spent with the line of the eyes below the tip of the shoulder
**Ear**	**Ear movements**	Both	Number of ear movements per minute
	**Ear on the sides**	Both	Proportion of time spent with the ears on both sides of the head (perpendicular to the head axis)
	Ear backwards	Both	Proportion of time spent with the ears orientated backwards
	**Ear forwards**	Both	Proportion of time spent with the ears orientated forwards
**Tail**	**Tail movements**	Both	Number of tail movements per minute
	**Tail high**	Both	Proportion of time spent with the tail base above the tip of the hindquarters
	**Tail low**	Both	Proportion of time spent with the tail base below the tip of the hindquarters
**Vocalizations**	Nicker	Equidae	Number of nickers per minute
	Whinny	Equidae	Number of whinnies per minute
	Squeal	Both	Number of squeals per minute
	**Grunt**	Suidae	Number of grunts per minute
	Scream	Suidae	Number of screams per minute
**Other behaviors**	Defecation	Both	Number of defecations per minute
	Foraging	Both	Proportion of time spent foraging on the floor
	**Eating**	Both	Proportion of time spent eating
	Negative interaction	Both	Interactions that triggered an avoidance behavior from the other animal
	Positive interaction	Both	Interactions that triggered an approach behavior toward the other animal

Blind-coding was achieved by watching the videos while unaware of the treatment and without the sound first, in order not to be influenced by the vocalizations played back. The videos were then watched a second time to record the vocalizations produced by the animals observed. Behaviors were scored either as occurrence (for discrete behaviors) or as duration (for continuous behaviors). We then divided these values by the total scoring time for each treatment (50 s), hence obtaining frequencies of occurrence for discrete behaviors (i.e., number of events per minute), and proportions of time spent performing the behavior for continuous behaviors. Analyses were carried out on these frequencies of occurrence or proportions.

We considered for the analyses only the behavioral parameters performed by at least 12 domestic horses and 12 Przewalski’s horses or 12 pigs and 10 wild boars (i.e., > 50% of the animals of each species; see parameters in bold in Table 5).

### Statistical analysis

All statistical analyses were performed with R software v.3.2.1 [47]. First, in order to eliminate redundancy due to the inter-correlation between the various scored behaviors and obtain composite scores for each response, hence avoiding multiple testing, we first carried out a principal component analysis (PCA; prcomp function, package stats) [48]. To be able to compare the results obtained for the wild and domestic species, for each Family (Equidae and Suidae), we combined the behavioral data collected on the domestic and wild species in the same PCA. For both Families, the first four principal components, which had an eigenvalue above one (Kaiser’s criterion; Tables 1 and 3), were extracted from the PCA (PC1–4) and used for further analyzes.

Then, for each species separately, the scores of these four PCs were entered as response variables in linear mixed-effect models (LMMs) fit with Gaussian family distribution and identity link function (lmer function, lme4 library in R), to test how they were affected by the species played in each session (1 species per session), the valence of each series of sounds played back in a given session (2 series of opposite valence in each session), and the valence of the first sound series played in each session (positive or negative depending on the session; Fig. 2A). These 16 models (one for each PC as an outcome variable and for each Family) included, as fixed factors, the species played (3 species: domestic horse, Przewalski’s horse and human; or pig, wild boar, and human), the valence of the sound series played back (positive or negative), the valence of the first sound series played in each session (positive valence followed by negative valence or vice versa; Fig. 2A), the sex-composition of individuals in the pair or group (only females, only males or mixed), and all possible two-way interactions terms between species played, valence, and the valence of the first sound series played. Finally, the test number (total = 30 tests in wild boars and 36 tests in other species, i.e., 10 (wild boars) or 12 (other species) groups or pair * 3 species played), nested within the identity of the animal tested, itself nested within the group or pair, was included as a random factor crossed with the session number (total = 3 for each pair or group, corresponding to the 3 species played), in order to account for dependencies between the data (i.e., same playback session, same animal, same pair or group). When an interaction term was significant, further post hoc tests were performed using Tukey’s honest significant difference (HSD).

The inclusion of non-significant interaction terms in models makes the interpretation of main effects problematic [49]. On the other hand, model simplification, in which non-significant terms including interactions are dropped from the full model, can lead to type 1 errors [50]. In order to be able to interpret main effects while leaving non-significant interactions in our models, we changed the contrasts of our factors (species played back, valence of the sound series, valence of the first sound series played, and gender-balance of the pair or group) from treatment contrasts (used by default by R) to sum contrasts [51].

For all models, we checked the residuals graphically for normal distribution and homoscedasticity. *P*-values (PBmodcomp function, package pbkrtest [52]) were calculated using parametric bootstrap methods (1000 bootstrap samples). *P*-values calculated with parametric bootstrap tests give the fraction of simulated likelihood ratio test statistic values (LRT) that are larger or equal to the observed LRT value [54]. Model estimates and confidence intervals were calculated for all models using a bootstrap approach (1000 samples, bootMer function, package lme4 [53]). All means are given with SDs.

## Supplementary information

**Additional file 1.** Data file containing the raw data.

## Data Availability

All data needed to evaluate the conclusions in the paper are present in the Additional file 1.
